# Fuzzy Cognitive Maps with Bird Swarm Intelligence Optimization-Based Remote Sensing Image Classification

**DOI:** 10.1155/2022/4063354

**Published:** 2022-03-27

**Authors:** Anwer Mustafa Hilal, Hadeel Alsolai, Fahd N. Al-Wesabi, Mohamed K Nour, Abdelwahed Motwakel, Anil Kumar, Ishfaq Yaseen, Abu Sarwar Zamani

**Affiliations:** ^1^Department of Computer and Self Development, Preparatory Year Deanship, Prince Sattam Bin Abdulaziz University, AlKharj, Saudi Arabia; ^2^Department of Information Systems, College of Computer and Information Sciences, Princess Nourah Bint Abdulrahman University, P.O. Box 84428, Riyadh 11671, Saudi Arabia; ^3^Department of Computer Science, College of Science & Art, Mahayil, King Khalid University, Saudi Arabia; ^4^Department of Computer Science, College of Computing and Information System, Umm Al-Qura University, Saudi Arabia; ^5^Data Science Research Group, School of Computing, DIT University, Dehradun, India

## Abstract

Remote sensing image (RSI) scene classification has become a hot research topic due to its applicability in different domains such as object recognition, land use classification, image retrieval, and surveillance. During RSI classification process, a class label will be allocated to every scene class based on the semantic details, which is significant in real-time applications such as mineral exploration, forestry, vegetation, weather, and oceanography. Deep learning (DL) approaches, particularly the convolutional neural network (CNN), have shown enhanced outcomes on the RSI classification process owing to the significant aspect of feature learning as well as reasoning. In this aspect, this study develops fuzzy cognitive maps with a bird swarm optimization-based RSI classification (FCMBS-RSIC) model. The proposed FCMBS-RSIC technique inherits the advantages of fuzzy logic (FL) and swarms intelligence (SI) concepts. In order to transform the RSI into a compatible format, preprocessing is carried out. Besides, the features are produced by the use of the RetinaNet model. Besides, a FCM-based classifier is involved to allocate proper class labels to the RSIs and the classification performance can be improved by the design of bird swarm algorithm (BSA). The performance validation of the FCMBS-RSIC technique takes place using benchmark open access datasets, and the experimental results reported the enhanced outcomes of the FCMBS-RSIC technique over its state-of-the-art approaches.

## 1. Introduction

With the advancement of Earth observation techniques, several kinds (for example, multi/hyperspectral and synthetic aperture radar) of higher-resolution images of Earth's surface are easily accessible [1]. Hence, it is highly significant to efficiently understand the semantic content, and more intelligent classification and identification techniques of land use and land cover (LULC) are certainly required. Remote sensing image (RSI) scene classification, which intends to automatically assign a certain semantic label to all the RSI scene patches based on its content, has become a hot topic in the fields of RSI interpretation due to its crucial application in land resource management, LULC, disaster monitoring, traffic control, and urban planning [2]. In recent times, various approaches were introduced for RSI scene classification [3].

The earlier method for scene classification have been largely dependent on lower-level or handcrafted features that aims at developing different human-engineering feature globally or locally, namely, texture, color, spatial, and shape data. A typical feature includes the color histogram (CH), scale invariant feature transform (SIFT), Gabor filters, local binary pattern (LBP), the histogram of oriented gradient (HOG), and gray level co-occurrence matrix (GLCM) are widely employed for scene classification [4]. It is noteworthy that methods based on this lower-level feature performed effectively on image with spatial arrangements or uniform texture, but still, they are constrained to distinguish images with more complex and challenging scenes, that is because the contribution of human in feature design considerably influence the efficiency of the representative capability of scene image [5]. In comparison with the lower-level feature-based method, the midlevel feature approach attempts to calculate a holistic image representation generated by local visual features including color histogram, SIFT, or LBP of the local image patch [6].

The common pipeline of constructing midlevel features is to extract local attributes of image patches initially and later for encoding them to attain the midlevel representation of RSI. The bag-of-visual-words (BoVW) method is one of the common midlevel methods and is broadly adapted for RSI scene classification due to its effectiveness and simplicity [7]. The method based on BoVW has enhanced performance of the classification; however, because of the limitations of representative ability of BOVW method, no other breakthrough has been accomplished for RSI scene classification. Recently, the deep learning (DL) method is commonly utilized in several image processes [8]. From the deep-restricted Boltzmann machines (DBM) and deep confidence networks (DBN) to deep convolution neural networks (CNN), significant improvement has been attained in distinct image fields. Particularly, CNN is acknowledged as one of the common techniques because of the capacity to learn hierarchical level abstraction of input data by encoded input data on distinct layers [2, 9]. In contrast to the conventional model, CNN approach has accomplished effective classification accuracy.

This study develops fuzzy cognitive maps with bird swarm optimization based RSI classification (FCMBS-RSIC) model. The proposed FCMBS-RSIC technique inherits the advantages of fuzzy logic (FL) and swarms intelligence (SI) concepts. In order to transform the RSI into a compatible format, pre-processing is carried out. Besides, the features are produced by the use of the RetinaNet model. Besides, a FCM-based classifier is involved to allocate proper class labels to the RSIs and the classification performance is enhanced by the design of bird swarm algorithm (BSA). The performance validation of the FCMBS-RSIC technique takes place using benchmark open access datasets.

## 2. Related Works

Zhang and others [10] presented an efficient RSI scene classification framework called CNN-CapsNet for using the advantages of these 2 techniques: CapsNet and CNN. First, a CNN without the FC layer is utilized as first feature map extractor. Particularly, a pretrained D-CNN method that has been completely trained on the ImageNet data set is carefully chosen as a feature extractor. Next, the first feature map is given to a recently developed CapsNet to attain the last classification outcome. Shawky and others [11] presented an effectual classification approach named CNN-MLP to use the merits of these 2 approaches: CNN and MLP. The feature is created by utilizing the pretrained CNN without a FC layer.

Li and others [12] introduced an RSSC-based error-tolerant deep learning (RSSC-ETDL) method for mitigating the negative effects of incorrect labels of the RSI scene datasets. In the presented approach, correcting error labels and learning multiview CNNs are simultaneously performed in an iterative method. It should be noticed that to generate the alternate system perform efficiently, we present an adoptive multifeature collaborative representation classification (AMF-CRC) which benefited from adoptively integrating various features of CNN for correcting the label of undefined sample. Xu and others [13] presented a classification model including RNN and RF for land classification with a satellite image that is open source for different study objectives. Then, the study utilized spatial data collected from the satellite image (that is time series).

Min and others [14] developed an approach called deep combinative feature learning (DCFL) for extracting lower-level texture and higher-level semantic data from various network layers. First, feature encoder VGGNet-16 is finetuned for succeeding multiscale feature extraction. Then, two shallow convolutions (Conv) layers are carefully chosen for convolution feature summing maps (CFSM), where we extract uniform LBP with rotation invariance for excavating comprehensive texture. A deep semantic feature from the FC layer concatenated with shallow feature constitutes deep combination feature that is thrown into SVM classification for last classification.

Huang and others [15] presented a task-adoptive embedding network for facilitating few-shot scene classification of RSI, represented as TAE-Net. First, a feature encoder was trained on the base set for learning embedded features of input image in the pretraining stage. Next, in the meta-training stage, a task-adoptive attention method was developed for producing the task-specific attention that could adoptively choose embedding features amongst the entire task. Yin and others [16] examined the fusion-based model for RSI scene classification from other viewpoints. First, it is classified into front, middle, and back side fusion modes. For every fusion mode, the correlated method is described and introduced. Next, classification performance of the single and hybrid side fusion modes is estimated.

## 3. Proposed Model

In this study, a new FCMBS-RSIC approach was developed for the detection and classification of RSIs. The proposed FCMBS-RSIC method encompasses distinct subprocesses such as pre-processing, RetinaNet-based feature extraction, FCM-based classification, and BSA-based parameter tuning. The design of BSA helps to properly tune the parameters involved in the FCM model, and consequently, the classification efficiency can be improved.

### 3.1. Preprocessing

Primarily, image pre-processing is carried out to make it compatible with further processes. Since the images are in the RGB format, they are transformed into grayscale versions. Besides, the unwanted portions of the images that are considered to be unwanted are removed. The images are filtered by the use of digital filters to get rid of the noise and discrepancies.

### 3.2. Feature Extraction: RetinaNet Model

At the time of feature extraction, the FCMBS-RSIC technique derives the feature vectors using the RetinaNet model. The CNNs are developed in an order of layers. An input map is stimulated with the individual's layer still achieving the resultant map [11]. Detailed individual layers are provided to demonstrate of computation equation. Let *X* ∈ *R*^*h*×*w*×*c*^(*h*: height, *w*: width, *c*: channel) are RGB images. All the layers get *X* and the group of parameters *W* as input and output a novel image *y* ∈ *R*^*h*′×*w*′×*c*′^, for instance, *y*=*f*(*X*,  *W*).

Primary, a convolution layer is an essential layer of the CNN. The learnable filter signifies the parameter of this layer sliding the filters on every input volume with existing width as well as height. This creates an activation map signifying the reaction of that filter at all spatial regions. In order to compute the convolutional of input *X* with bank of filters $W\in R^{\bar{h}x\bar{w}\times \bar{c}\times c^{\prime }}$ and adding a bias ∈*R*^*c*′^, equation (1) was utilized.

(1) $$\begin{array}{c} y_{i^{\prime }j^{\prime }k^{\prime }}=f\left(b_{k^{\prime }}+\sum_{i=1}^{\bar{h}}\sum_{j=1}^{\bar{w}}\sum_{d=1}^{c}W_{ij\;dk}\times X_{i^{\prime }+i,\;j^{\prime }+j,d^{\prime }}\right). \end{array}$$

Second, the max‐pooling layer was utilized for decreasing the parameter and computation from the network with decreasing the size of imputing shapes. It calculates the maximal response of all image channels from $\tilde{h}\times \tilde{w}$ sub window that performs as subsampling function. It is formulated as follows:

(2) $$\begin{array}{c} y_{i^{\prime }j^{\prime }k^{\prime }=\underset{1<i<\tilde{h}}{\max},\;1<j<\tilde{w}}X_{i^{\prime }+ij^{\prime }+j,\;k}. \end{array}$$

Eventually, FC layers are a group of layers that combine the data extracting by preceding layer (feature). These layers get an input *X*, process it, and the final FC layer creates 1D vector of size equivalent the amount of classes.

RetinaNet mostly comprises 3 subnetworks [17] as ResNet, feature pyramid network (FPN), and 2 FCNs. The essential support of ResNet is the knowledge of residual learning that permits the novel input data that is directly transferred to the subsequent layer. The ResNet utilizes various network layers. The generally utilized kinds of network layers are 50_layer, 101_layer, and 152_layer. The 101_layer framework with optimum trained efficiency can be selected. It can remove the structures of echocardiography utilizing ResNet and afterward put those away to next sub-network. An FPN is a technique to effectively remove the feature of all dimensions from picture utilizing a convention CNN technique.

**Figure 1:**
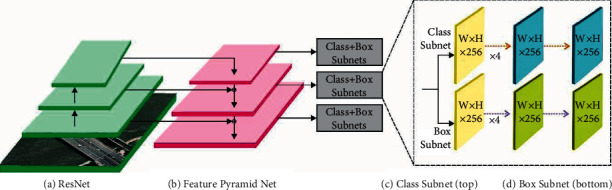
Structure of RetinaNet [17].

Primarily, a single dimension image can be utilized as the input to ResNet. Next, based on the secondary layer of convolution network, the feature of every layer is chosen by FPN and then integrate for creating the last feature output combinations. The class subnet from the FCN carried out the classification task. This subnet is recognized that view the echocardiography image appears to. The box subnet from the FCN carried out the border regression tasks. Its role is for detecting the place of left ventricle from the echocardiography image and recording the co-ordinate.  Figure 1 demonstrates the framework of RetinaNet.

Focal loss: the focal loss is an enhanced form of cross entropy (CE) loss, and the binary CE expression is as follows:

(3) $$\begin{array}{c} CE\left(p,\;y\right)=\left\{\begin{array}{cc} -\text{ log }\left(p\right), & \text{if }y=1,\\ -\text{ log }\left(1-p\right), & \text{otherwise,} \end{array}\right) \end{array}$$

where *y* ∈ [±,  1] signifies the ground truth type and *p* ∈ [0,1] indicates the forecast probabilities of model to type *y*=1.

(4) $$\begin{array}{c} p_{t}=\left\{\begin{array}{cc} p, & \text{if }y=1,\\ 1-p, & \text{otherwise}. \end{array}\right) \end{array}$$

The previous equation is abbreviated as follows:

(5) $$\begin{array}{c} CE\left(p,\;y\right)=CE\left(p_{t}\right)\\ =-\text{ log }\left(p_{t}\right). \end{array}$$

For solving the issue of data imbalance amongst the positive as well as negative instances, the novel procedure was altered as to the subsequent method:

(6) $$\begin{array}{c} CE\left(p_{t}\right)=-\alpha_{t}\text{ log }\left(p_{t}\right). \end{array}$$

Amongst them,

(7) $$\begin{array}{c} \alpha_{t}=\left\{\begin{array}{cc} \alpha , & \text{if }y=1,\\ l-\alpha , & \text{otherwise,} \end{array}\right) \end{array}$$

where *α* ∈ [0,1] refers the weight factors. For solving the issue of complex instance, the concentrating parameter *C* was established for obtaining the last procedure of focal loss:

(8) $$\begin{array}{c} FL\left(p_{t}\right)=-\alpha_{t}{\left(1-p_{t}\right)}^{\gamma }\text{ log }\left(p_{t}\right). \end{array}$$

### 3.3. Image Classification: FCM Model

**Figure 2:**
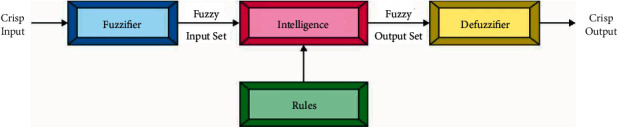
Process of fuzzy logic.

During classification process, the feature vectors are passed into the FCM model to allot class labels. FCM could be viewed as RNN using interpretability features that were commonly utilized in modeling tasks [18]. They comprise a collection of neural processing entities named concept (neuron) and the causal relation. The activation values of this neuron commonly take values within [0,1], hence the strong the activation values, the great its effect on the system. Obviously, connected weight is also applicable in this system. The power of casual relations among two neurons *C*_*i*_ and *C*_*j*_ is quantified by arithmetical weight *w*_*ij*_ ∈ [−1,1] and represented as a causal edge from *C*_*i*_ to $C_{\dot{j}}.$Figure 2 illustrates the process flow of FL. There are three potential kinds of causal relations among neural processing units in the FCM‐based network that express the kind of impact from one neuron to another that is given in the following list:

- When *w*_*ij*_ > 0, a rise (decrement) in the cause *C*_*i*_ produces an increment (decrement) of the impact *C*_*j*_ with intensity |*w*_*ij*_|.
- When *w*_*ij*_ < 0, a rise (decrement) in the cause *C*_*i*_ produces a decrement (increment) of the neuron *C*_*j*_ with intensity |*w*_*ij*_|.
- When *w*_*ij*_=0, there are no causal relations among *C*_*i*_ and *C*_*j*_.  This rule is iterated till an ending criterion is satisfied. A new activation vector is estimated at every step *t* and afterward a fixed amount of iterations. The FCM is stated to have converged when it reaches fixed‐point attractors or else the update procedure stops afterward a maximal amount of iterations *T* is attained.

(9) $$\begin{array}{c} A_{i}^{\left(t+1\right)}=f\left(\sum_{j=1}^{M}w_{ji}A_{j}^{\left(t\right)}\right),\;i\neq j. \end{array}$$

The function *f*(·) signifies a monotonically nonreducing nonlinear function utilized for clamping the activation values of all the neurons to the interval. An instance of this function is the sigmoid variants, bivalent function, and trivalent function. Then, attention is drawn toward the sigmoid function because it has displayed greater predictive abilities A nonlinear transfer function is utilized in the study, whereas *λ* represents the sigmoid slope and *h* indicates the offset. Various researches have revealed that this parameter is tightly linked to network convergence.

(10) $$\begin{array}{c} f\left(A_{i}\right)=\frac{1}{1+e^{-\lambda \left(A_{i}-h\right)}}. \end{array}$$

This rule is chosen while upgrading the activation value of neuron which is not impacted by neural processing entity.

(11) $$\begin{array}{c} A_{i}^{\left(t+1\right)}=f\left(\sum_{j=1}^{M}w_{ji}A_{j}^{\left(t\right)}+A_{i}^{\left(t\right)}\right),\;i\neq j. \end{array}$$

The alternative adapted upgrading rule has been presented for avoiding the conflict that emerges in the event of nonactive neuron. More apparently, the rescaled inference permits handling the scenario while there are no data regarding a first neuron state and assist to prevent the saturation issue.

(12) $$\begin{array}{c} A_{i}^{\left(t+1\right)}=f\left(\sum_{j=1}^{M}w_{ji}\left(2A_{j}^{\left(t\right)}-1\right)+\left(2A_{i}^{\left(t\right)}-1\right)\right),\;i\neq j. \end{array}$$

When the cognitive network is capable of converging, the scheme would generate the similar output, and then the activation degree of neuron remains unchanged. At the same time, a cyclic FCM generates different responses with the exception of some state that is regularly generated. The final potential scenarios are associated with chaotic configuration where the network produces distinct state vectors.

### 3.4. Parameter Optimization: Bird Swarm Algorithm

In order to optimally adjust the parameters involved in the FCM technique, the BSA is applied to it. BSA, presented by Meng and others [19], is a novel intelligent bionic technique dependent upon multigroup and multisearch techniques; it simulates the birds foraging performance, vigilance performance, and flight performance, and utilizes this SI for solving the optimized issue. The bird's swarm technique was based on 5 rules:

**Rule 1 .**

All the birds are switching amongst vigilant as well as foraging performance, and combined bird forage and keep vigilance are simulated as arbitrary decisions.

**Rule 2 .**

if the foraging, all birds recorded and updated their preceding optimum knowledge and swarm prior optimum skill with food patch. The skill is also be utilized for searching for food. Instant sharing of social data was through the group.

**Rule 3 .**

Once they keep vigilance, all birds attempts for moving near the center of swarms. It is performance may be controlled by disturbance due to swarm competition. The bird with further stocks was highly possible toward swarm centers than bird with lease stock.

**Rule 4 .**

The bird flies to another location frequently. If flying to another place, birds frequently switch amongst production as well as shrub. The bird with maximum stocks are producers, and bird with minimum is scrounger. Another bird with maximal and minimal reserves is arbitrarily chosen to producer and scrounger.

**Rule 5 .**

Producer actively seeks food. The scroungers arbitrarily follow producers searching for food.

Based on Rule 1, it can be determined that the time interval of all birds flight performance *FQ*, the probabilities of foraging performance *P*(*P* ∈ (0,1 and uniform arbitrary number *δ* ∈ (0,1))).

When the amount of iteration was lesser than FQ and *δ* ≤ *P*, the bird was foraging performance.  Rule 2 is formulated mathematically as follows:

(13) $$\begin{array}{c} x_{i,j}^{t+1}=x_{i,j}^{t}+\left(p_{i,j}^{t}-x_{i,j}^{t}\right)\times C\times \text{ rand }\left(0,1\right)+\left(g_{j}^{t}-x_{i,j}^{t}\right)\times S\times \text{ rand }\left(0,1\right), \end{array}$$

where *C* and *S* are 2 positive numbers; the previous is named as the cognitive accelerated co-efficient, and the final is named as the social accelerated co-efficient. At this point, *p*_*i*,*j*_ represents the *i*^*th*^ bird optimum preceding place and *g*_*j*_ signifies the optimum previous swarm place [20].

When the amount of iteration is lesser than FQ and *δ* > *P*, the bird is vigilance performance.  The Rule 3 is formulated mathematically as follows:

(14) $$\begin{array}{c} x_{i,j}^{t+1}=x_{i,j}^{t}+A_{1}\left({\text{mean}}_{j}^{t}-x_{i,j}^{t}\right)\times \text{ rand}\left(0,1\right)+A_{2}\left(p_{k,j}^{t}-x_{i,j}^{t}\right)\times \text{ rand }\left(-1,1\right),\\ A_{1}=a_{1}\times \text{ exp }\left(-\frac{pFit_{i}}{\text{sumFit}+\varepsilon }\times N\right),\\ A_{2}=a_{2}\times \text{ exp }\left(\left(\frac{p{\text{Fit}}_{i}-p{\text{Fit}}_{k}}{\left|p{\text{Fit}}_{k}-p{\text{Fit}}_{i}\right|+\varepsilon }\right)\times \frac{N\times p{\text{Fit}}_{k}}{\text{sumFit}+\varepsilon }\right), \end{array}$$

where *a*_1_ and *a*_2_ denotes the 2 positive constants from zero and two, *p*Fit_*i*_ indicates the optimum fitness value of *i*^*th*^ bird and sumFit refers to the sum of swarms' optimum fitness value. At this point, *ε* that are utilized for avoiding zero‐division error is the minimum constant from the computer. mean_*j*_ stands for the *j*^*th*^ element of entire swarm's average place.

When the amount of iteration is equivalent *FQ*, the bird is flight performance that is separated as to performance of producer and scrounger by fitness.  Rule 3 and Rule 4 are formulated mathematically as

(15) $$\begin{array}{c} x_{i,j}^{t+1}=x_{i,j}^{t}+\text{rand}n\;\left(0,1\right)\times x_{i,j}^{t},\;\\ x_{i,j}^{t+1}=x_{i,j}^{t}+\left(x_{k,j}^{t}-x_{i,j}^{t}\right)\times FL\times \text{rand }\left(0,1\right), \end{array}$$

where FL (*FL* ∈ [0,2]) demonstrates that the scrounger is follow the producers for searching for food.

The BSA approach derives a FF for reaching increased classification efficiency. It resolves a positive integer for representing the optimum efficiency of the candidate solution. During this case, the minimized classifier error rate was assumed that FF is provided in equation (16). The optimal result is a lower error rate and worst solution gains an enhanced error rate.

(16) $$\begin{array}{c} \text{fitness}\left(x_{i}\right)=\text{ClassifierErrorRate}\left(x_{i}\right)\\ =\frac{\text{number of misclassified instances}}{\text{total number of instances}}*100. \end{array}$$

## 4. Experimental Validation

**Figure 3:**
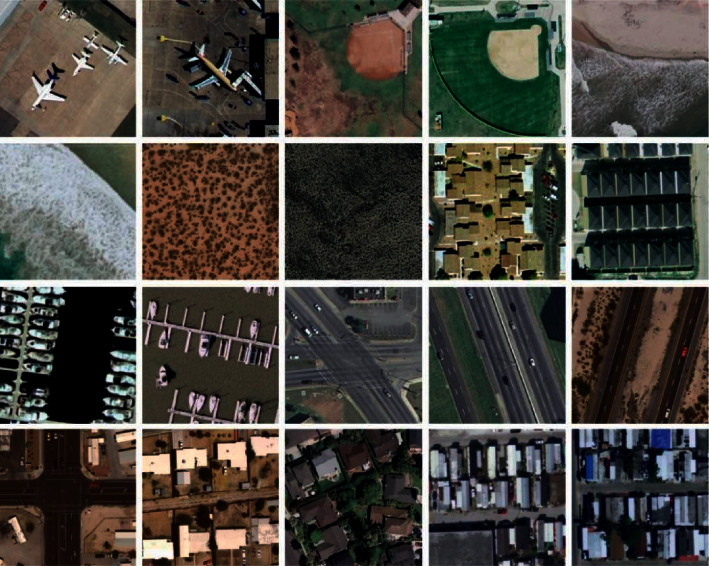
Sample images UCM21 dataset.

**Figure 4:**
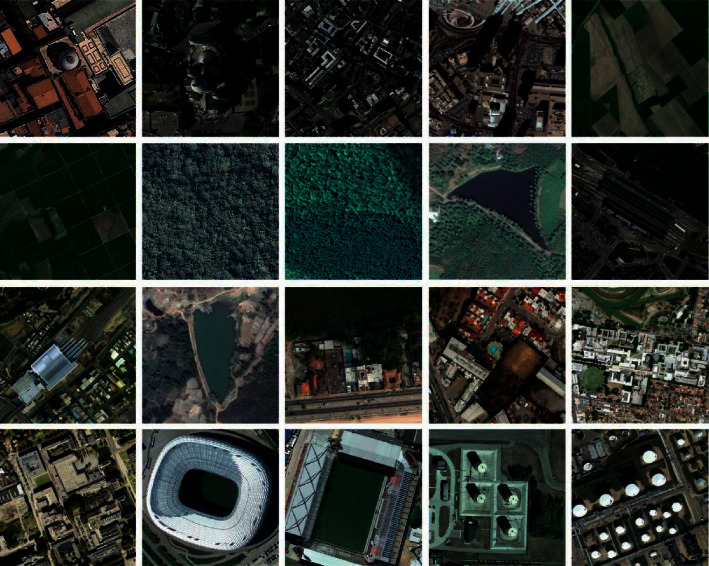
Sample images AID dataset.

The simulation of the FCMBS-RSIC technique is performed using a Python 3.6.5 tool. The experimental result analysis of the FCMBS-RSIC technique is validated using two benchmark datasets, namely, UCM21 [21] and AID [22] datasets. The UCM dataset contains images under 21 classes with a set of 100 images under every class. The size of the images in the dataset is 256 ^∗^ 256 pixels. Besides, the AID dataset includes 30 classes with 10K images under each class.  Figure 3 and Figure 4 illustrates the sample images of two datasets. The parameter setting of the proposed model is given as follows. Batch size: 500, max. Epochs:15, learning rate: 0.05, dropout rate: 0.2, and momentum: 0.9. The proposed model is simulated using Processor - i5-8600k, Graphics Card - GeForce 1050Ti 4 GB, 16 GB RAM, and OS Storage - 250 GB SSD.

**Figure 5:**
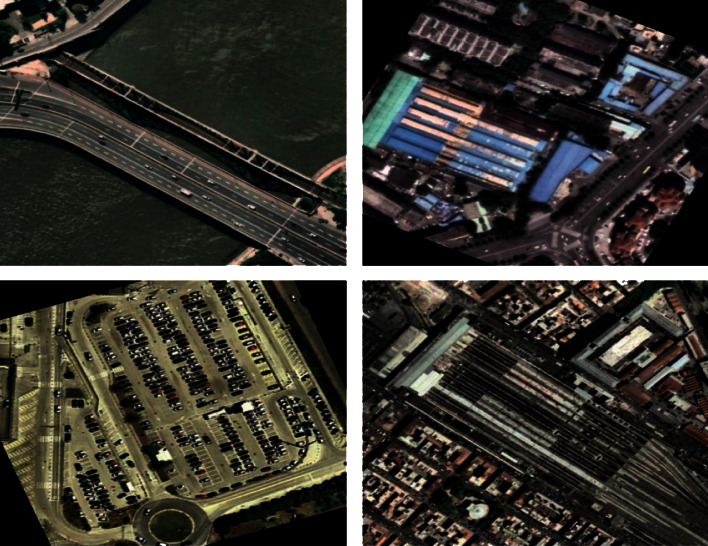
Preprocessed images.

**Figure 6:**
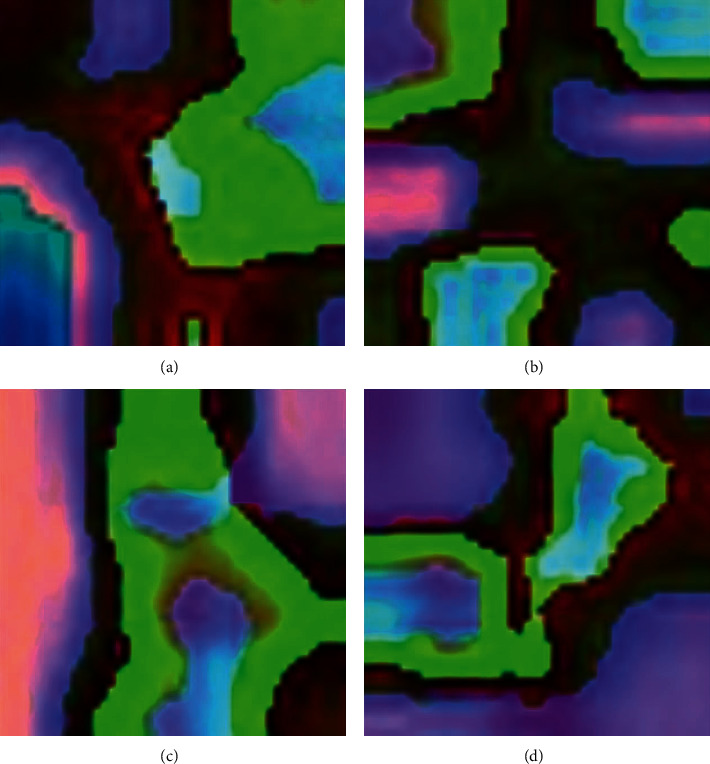
Feature Maps. (a) Airport. (b) Bare land. (c) Beach. (d) Bridge.

Figure 5 illustrates the preprocessed version of the test RSI by the FCMBS-RSIC technique. The figures reported that the image quality gets improved and it helps to increase the classification outcomes of the FCMBS-RSIC technique.  Figure 6 illustrates the feature maps obtained by the FCMBS-RSIC technique on four test images namely airport, bare land, beach, and bridge.

A comprehensive classification result analysis of the FCMBS-RSIC technique under varying sizes of training/testing data of UCM21 dataset is offered in Table 1.

**Figure 7:**
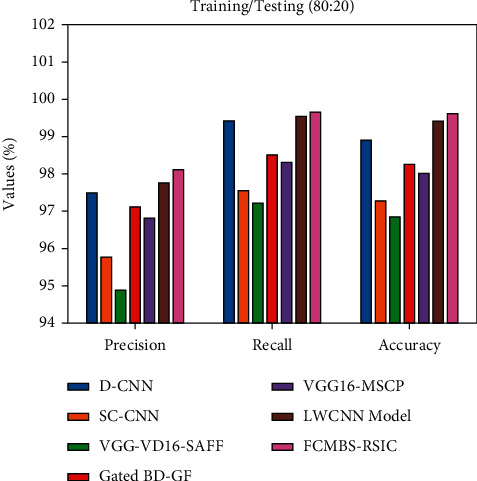
Comparative analysis of the FCMBS-RSIC technique under training/testing (80 : 20) data of the UCM21 dataset.

Figure 7 examines the comparison study of the FCMBS-RSIC technique with recent methods [23] under training/testing (80 : 20) data of UCM21 dataset. The experimental results revealed that the D-CNN, SC-CNN, and VGG-VD16-SAFF techniques have gained ineffective outcomes with the least values of prec_*n*_, reca_*l*_, and accu_*y*_. Also, the gated BD-GF and VGG16-MSCP techniques have attained slightly raised values of prec_*n*_, reca_*l*_, and accu_*y*_. In addition, the LWCNN technique has gained somewhat reasonable outcome with the *prec*_*n*_, *reca*_*l*_, and *accu*_*y*_ of 97.76%, 99.55%, and 99.42%, respectively. However, the FCMBS-RSIC technique has shown better results with the prec_*n*_, reca_*l*_, and accu_*y*_ of 98.12%, 99.67%, and 99.63%, respectively.

**Figure 8:**
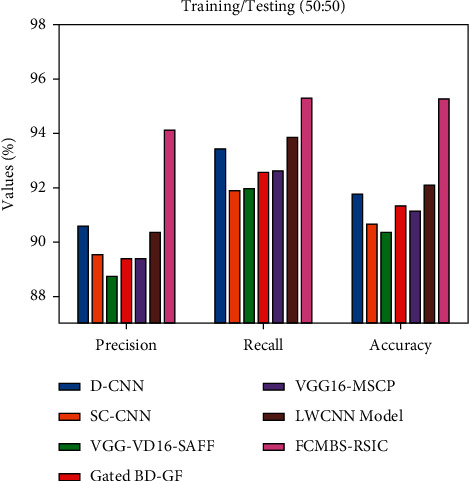
Comparative analysis of the FCMBS-RSIC technique under training/testing (50 : 50) data of the UCM21 dataset.

Figure 8 illustrates the performance analysis of the FCMBS-RSIC technique with existing techniques under training/testing (50 : 50) data of the UCM21 dataset. The results indicated that the D-CNN, SC-CNN, and VGG-VD16-SAFF techniques have attained lower values of prec_*n*_, reca_*l*_, and accu_*y*_. Concurrently, the gated BD-GF and VGG16-MSCP techniques have resulted in somewhat improved values of prec_*n*_, reca_*l*_, and accu_*y*_. Simultaneously, the LWCNN technique has demonstrated considerable performance with the prec_*n*_, reca_*l*_, and accu_*y*_ of 90.35%, 93.83%, and 92.10%. However, the FCMBS-RSIC technique has gained maximum performance with the prec_*n*_, reca_*l*_, and accu_*y*_ of 94.12%, 95.32%, and 95.27%, respectively.

**Figure 9:**
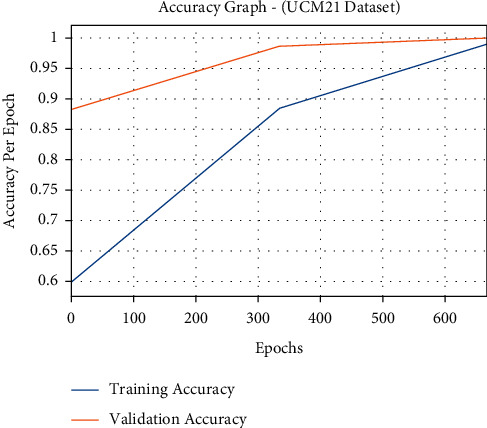
Accuracy analysis of FCMBS-RSIC technique on the UCM21 dataset.

The accuracy outcome analysis of the FCMBS-RSIC technique on UCM21 dataset is portrayed in Figure 9. The results demonstrated that the FCMBS-RSIC approach has accomplished higher validation accuracy compared to training accuracy. It is also observable that the accuracy values get saturated with the count of epoch.

**Figure 10:**
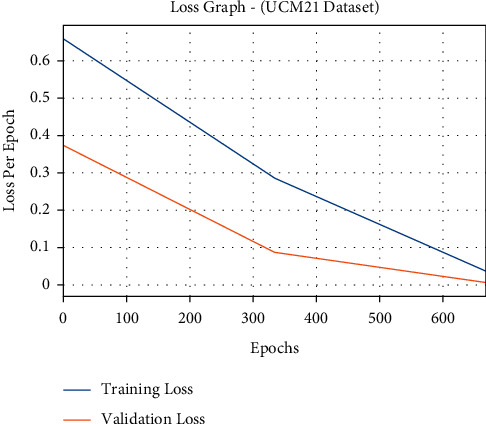
Loss analysis of FCMBS-RSIC technique on the UCM21 dataset.

The loss outcome analysis of the FCMBS-RSIC technique on UCM21 dataset is illustrated in Figure 10. The figure exposed that the FCMBS-RSIC system has denoted the reduced validation loss over the training loss. It is additionally noticed that the loss values get saturated with the count of epoch.

Table 2 provides the RSI classification result analysis of the FCMBS-RSIC technique under different sizes of training/testing data of the AID dataset.

**Figure 11:**
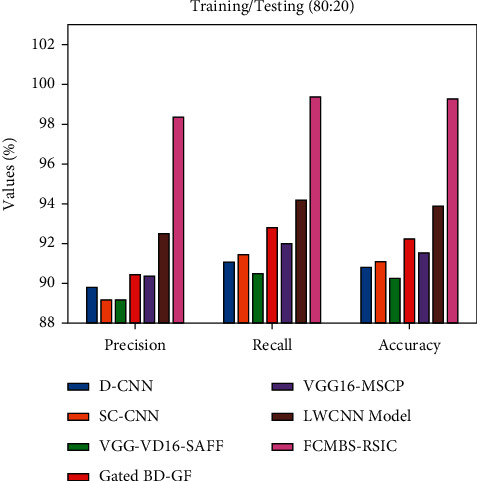
Comparative analysis of the FCMBS-RSIC technique under training/testing (80 : 20) data of the AID dataset.

Figure 11 inspects the classifier result analysis of the FCMBS-RSIC technique with recent methods under training/testing (80 : 20) data of AID dataset. The results indicated that the D-CNN, SC-CNN, and VGG-VD16-SAFF techniques have accomplished worse outcomes with the lower values of prec_*n*_, reca_*l*_, and *accu*_*y*_. Besides, the gated BD-GF and VGG16-MSCP techniques have provided certainly increased values of prec_*n*_, reca_*l*_, and accu_*y*_. The LWCNN technique has exhibited competitive outcome with the prec_*n*_, reca_*l*_, and *accu*_*y*_ of 92.51%, 94.18%, and 93.85%. However, the FCMBS-RSIC technique has shown better results with the prec_*n*_, reca_*l*_, and accu_*y*_ of 98.36%, 99.42%, and 99.31%, respectively.

**Figure 12:**
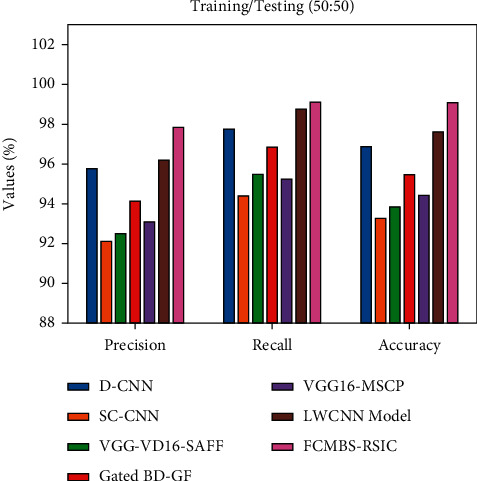
Comparative analysis of the FCMBS-RSIC technique under training/testing (80 : 20) data of AID dataset.

Figure 12 reports the comparative result analysis of the FCMBS-RSIC technique with existing techniques under training/testing (50 : 50) data of the AID dataset. The table values revealed that the D-CNN, SC-CNN, and VGG-VD16-SAFF techniques have exhibited poor performance with the minimum values of prec_*n*_, reca_*l*_, and accu_*y*_.

Eventually, the gated BD-GF and VGG16-MSCP techniques have resulted in somewhat improved values of prec_*n*_, reca_*l*_, and accu_*y*_. Meanwhile, the LWCNN technique has demonstrated considerable performance with the prec_*n*_, reca_*l*_, and accu_*y*_ of 96.22%, 98.75%, and 97.64%. However, the FCMBS-RSIC technique has presented effective outcomes with the prec_*n*_, reca_*l*_, and accu_*y*_ of 97.86%, 99.12%, and 99.06%, respectively.

**Figure 13:**
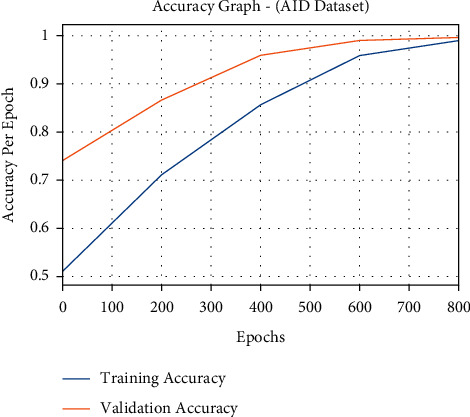
Accuracy analysis of the FCMBS-RSIC technique on AID dataset.

The accuracy outcome analysis of the FCMBS-RSIC method on AID dataset is showcased in Figure 13. The outcomes outperformed that the FCMBS-RSIC system has accomplished maximum validation accuracy compared to training accuracy. It is also observable that the accuracy values get saturated with the count of epoch.

**Figure 14:**
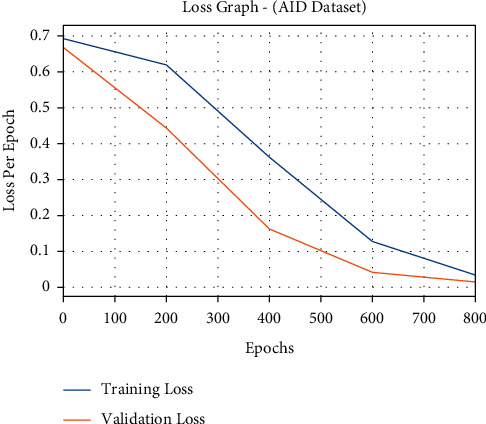
Loss analysis of FCMBS-RSIC technique on the AID dataset.

The loss outcome analysis of the FCMBS-RSIC methodology on AID dataset is demonstrated in Figure 14. The figure is obvious that the FCMBS-RSIC technique has referred to the lower validation loss over the training loss. It can be additionally noticed that the loss values get saturated with the count of epoch.

**Figure 15:**
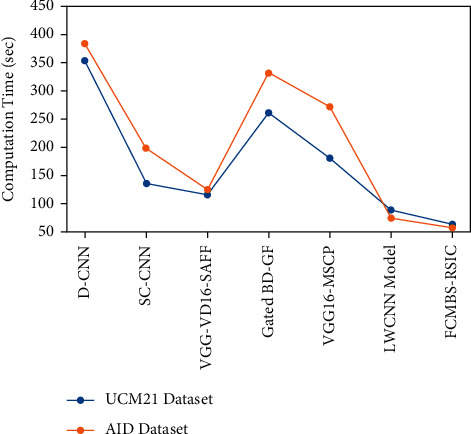
CT analysis of FCMBS-RSIC technique with existing methods.

Lastly, a detailed computation time (CT) analysis of the FCMBS-RSIC technique on the test UCM21 and AID datasets is given in Table 3 and Figure 15. The experimental values indicated that the D-CNN model has shown ineffective results with the maximum CT on the test datasets. In addition, the gated BD-GF and VGG16-MSCP techniques have resulted in slightly reduced CT over the D-CNN technique.

Along with that, the SC-CNN and VGG-VD16-SAFF techniques have reached moderately closer CT. Though the LWCNN technique has attained reasonable CT of 89s and 74s on the UCM21 and AID datasets, the proposed FCMBS-RSIC technique has outperformed the other methods with the lower CT of 64s and 58s, respectively. By looking into the above mentioned tables and figures, it is ensured that the FCMBS-RSIC technique has the ability of effectually classify RSIs.

## 5. Conclusion

In this study, a new FCMBS-RSIC methodology was developed for the detection and classification of RSIs. The proposed FCMBS-RSIC method encompasses different subprocesses such as preprocessing, RetinaNet-based feature extraction, FCM-based classification, and BSA-based parameter tuning. The design of BSA helps to properly tune the parameters contained in the FCM model, and consequently, the classification efficiency can be improved. The performance validation of the FCMBS-RSIC technique takes place using benchmark open access datasets and the results are examined under several aspects. The comparative experimental outcomes described the enhanced outcomes of the FCMBS-RSIC method over its recent approaches. Therefore, the FCMBS-RSIC technique can be treated as an effective tool for RSI classification. In future, hybrid DL models can be derived to improve the classifier results of the FCMBS-RSIC technique.

## Figures and Tables

**Table 1:** Comparative analysis of the FCMBS-RSIC technique with existing approaches under the UCM21 dataset.

Methods	Training/testing (80 : 20)			Training/testing (50 : 50)		
	Precision	Recall	Accuracy	Precision	Recall	Accuracy
D-CNN	97.50	99.44	98.92	90.58	93.44	91.78
SC-CNN	95.76	97.55	97.28	89.54	91.88	90.65
VGG-VD16-SAFF	94.90	97.22	96.86	88.71	91.96	90.37
Gated BD-GF	97.11	98.53	98.26	89.37	92.56	91.34
VGG16-MSCP	96.82	98.33	98.03	89.39	92.62	91.15
LWCNN model	97.76	99.55	99.42	90.35	93.83	92.10
FCMBS-RSIC	98.12	99.67	99.63	94.12	95.32	95.27

**Table 2:** Comparative analysis of the FCMBS-RSIC technique with existing approaches under the AID dataset.

Methods	Training/testing (80 : 20)			Training/testing (50 : 50)		
	Precision	Recall	Accuracy	Precision	Recall	Accuracy
D-CNN	89.81	91.10	90.82	95.78	97.77	96.89
SC-CNN	89.15	91.48	91.10	92.12	94.41	93.30
VGG-VD16-SAFF	89.18	90.49	90.25	92.51	95.46	93.83
Gated BD-GF	90.43	92.78	92.20	94.18	96.85	95.48
VGG16-MSCP	90.36	92.00	91.52	93.11	95.24	94.42
LWCNN model	92.51	94.18	93.85	96.22	98.75	97.64
FCMBS-RSIC	98.36	99.42	99.31	97.86	99.12	99.06

**Table 3:** Computation time analysis of FCMBS-RSIC technique under two datasets.

Computation time (sec)		
Methods	UCM21 dataset	AID dataset
D-CNN	354	383
SC-CNN	136	198
VGG-VD16-SAFF	116	125
Gated BD-GF	262	332
VGG16-MSCP	181	272
LWCNN model	089	074
FCMBS-RSIC	064	058

## Data Availability

Data sharing is not applicable to this article as no datasets were generated during this study.
